# Effect of chiropractic treatment on primary or early secondary prevention: a systematic review with a pedagogic approach

**DOI:** 10.1186/s12998-018-0179-x

**Published:** 2018-04-05

**Authors:** Guillaume Goncalves, Christine Le Scanff, Charlotte Leboeuf-Yde

**Affiliations:** 1 CIAMS, University of Paris-Sud, University of Paris-Saclay, F-91405 Orsay Cedex, France; 2 CIAMS, University of Orléans, F-45067 Orléans, France; 3 Institut Franco Européen de Chiropraxie, 24 boulevard Paul Vaillant Couturier, F-94200 Ivry sur Seine, France

**Keywords:** Chiropractic, Primary prevention, Early secondary prevention, Public health, Chiropraxie, Prévention primaire, Prévention secondaire précoce, Santé publique

## Abstract

**Introduction:**

The chiropractic vitalistic approach to the concept of ‘subluxation’ as a cause of disease lacks both biological plausibility and possibly proof of validity. Nonetheless, some chiropractors purport to prevent disease in general through the use of chiropractic care. Evidence of its effect is needed to be allowed to continue this practice. The objective of this systematic review was therefore to investigate if there is any evidence that spinal manipulations/chiropractic care can be used in primary prevention (PP) and/or early secondary prevention in diseases other than musculoskeletal conditions.

**Method:**

We searched *PubMed, Embase, Index to Chiropractic Literature*, and some specialized chiropractic journals, from inception to October 2017, using terms including: “chiropractic”, “subluxation”, “wellness”, “prevention”, “spinal manipulation”, “mortality”. Included were English language articles that indicated that they studied the clinical preventive *effec*t *of* or *benefit from* manipulative therapy/chiropractic treatment in relation to PP and/or early treatment of physical diseases/morbidity in general, other than musculoskeletal disorders. Also, population studies were eligible. Checklists were designed in relation to the description of the reviewed articles and some basic quality criteria. Outcomes of studies were related to their methodological quality, disregarding results from those unable to answer the research questions on effect of treatment.

**Results:**

Of the 13.099 titles scrutinized, 13 articles were included (eight clinical studies and five population studies). These studies dealt with various disorders of public health importance such as diastolic blood pressure, blood test immunological markers, and mortality. Only two clinical studies could be used for data synthesis. None showed any effect of spinal manipulation/chiropractic treatment.

**Conclusion:**

We found no evidence in the literature of an effect of chiropractic treatment in the scope of PP or early secondary prevention for disease in general. Chiropractors have to assume their role as evidence-based clinicians and the leaders of the profession must accept that it is harmful to the profession to imply a public health importance in relation to the prevention of such diseases through manipulative therapy/chiropractic treatment.

## Abstract (fr)

**Introduction:**

Une partie des chiropracteurs ayant une conception vitaliste fondent leur approche sur le diagnostic et le traitement des « subluxations ». Dans cette optique, ces dernières pourraient être à l’origine de troubles non musculo squelettiques. Ce postulat n’est. pas plausible et semble manquer de validité. Malgré cela, certains chiropracteurs proposent de prévenir certaines maladies à travers des soins chiropratiques. L’objectif majeur de cet article est. de recenser les preuves concernant la prévention primaire (PP) ou la prévention secondaire précoce des maladies (autres que les troubles neuro musculo squelettiques) en chiropraxie.

**Méthode:**

Une recherche bibliographique a été effectuée jusqu'en octobre 2017 via *PubMed, Embase, Index to Chiropractic Literature*, et d’autres journaux chiropratiques spécialisés. Les termes suivants ont été utilisés: *« chiropractic », « subluxation », « wellness », « prevention », « spinal manipulation », « mortality »*. Les articles publiés en anglais et indiquant étudier les effets ou bénéfices des thérapies manuelles/de la chiropraxie dans le cadre de la PP/de la prévention secondaire précoce des maladies et/ou de la morbidité en générale ont été inclus, ainsi que les études effectuées sur des registres de populations. Des *checklists* ont été créées afin de décrire et de critiquer de manière simplifiée la qualité des études. Les résultats des études ont été pris en compte en fonction de leur qualité méthodologique. Ceux provenant des études ayant un design non adapté aux questions de recherche concernant les effets ou bénéfices du traitement chiropratique n’ont pas été pris en compte.

**Résultats:**

Sur les 13,099 titres recensés, 13 articles ont été retenus (huit études cliniques et cinq études sur des registres de populations). Ces études traitent de différents sujets d’importance en santé publique: hypertension artérielle, troubles immunologiques, mortalité, etc. Seulement deux études ont pu être utilisées pour l’analyse de leurs résultats. Aucunes d’entre elles n’objectivent un effet suite aux manipulations vertébrales/ traitements chiropratiques.

**Conclusion:**

Aucune preuve dans la littérature sur l’effet ou bénéfice des manipulations vertébrales/traitements chiropratiques dans le cadre de la PP ou de la prévention secondaire précoce des maladies en général n’a pu être mis en évidence. Les chiropracteurs doivent adopter une pratique *evidence based*, et les dirigeants de la profession devraient accepter qu’il soit dangereux d’affirmer avoir un rôle dans la prévention des maladies non musculo-squelettiques par la thérapie manuelle.

## Introduction

### Primary and early prevention

From a public health perspective, it is always relevant to consider whether it is possible to prevent a disease from occurring rather than to treat it when it is present. However, primary prevention (PP) or early secondary prevention is not always successful or suitable. This can only be applied to conditions with known and avoidable causes. There are also other obvious ‘rules’ for applying a preventive approach, such as it having to be both less costly and less likely to cause adverse effects than the treatment.

### Primary prevention in chiropractic practice

Some medical conditions are well documented for being successfully prevented through PP or early secondary prevention, such as polio through vaccination [1] and dental caries through oral hygiene and fluoride exposure [2]. According to a recent systematic review, chiropractors are interested in providing PP both in relation to several general public health issues and for musculoskeletal conditions [3]. In fact, some chiropractors have adopted the ‘dental model’ in their practice, proposing to prevent spinal problems through treatment of spinal subluxations before symptoms arise although patients do not seem to be particularly interested in this aspect of chiropractic care [3]. In addition, some chiropractors even offer to help preventing disease in general and advocate their services also to people who wish to maintain good health, basing this on a ‘vitalist’ approach which includes a belief in the ‘subluxation concept’ [4, 5]. This alternative approach offers chiropractic care (possibly in conjunction with other modalities if needed) to prevent disease in general also for disorders outside the usual chiropractic scope of practice and is therefore sometimes beyond the legal boundaries for chiropractic legislation [6].

The definition of absence of disease, for this group of chiropractors, could be the World Health Organisation definition of health, as “a state of complete physical, mental and social well-being and not merely the absence of disease or infirmity” [7]. In order to obtain this complete healthy state, according to proponents of this approach, chiropractic treatment offers some considerable possibilities, because, according to Hannon in a review of the chiropractic literature: “It is plausible that chiropractic care may be of benefit to every function of the body and have the potential for long-term, overall health benefit to those receiving chiropractic care” [8].

### Challenging the subluxation model

Beliefs that a spinal subluxation can cause a multitude of diseases and that its removal can prevent them is clearly at odds with present-day concepts, as the aetiology of most diseases today is considered to be multi-causal, rarely mono-causal. It therefore seems naïve when chiropractors attempt to control the combined effects of environmental, social, biological including genetic as well as noxious lifestyle factors through the simple treatment of the spine. In addition, there is presently no obvious emphasis on the spine and the peripheral nervous system as the governing organ in relation to most pathologies of the human body.

The ‘subluxation model’ can be summarized through several concepts, each with its obvious weakness. According to the first three, (i) disturbances in the spine (frequently called ‘subluxations’) exist and (ii) these can cause a multitude of diseases. (iii) These subluxations can be detected in a chiropractic examination, even before symptoms arise. However, to date, the subluxation has been elusive, as there is no proof for its existence. Statements that there is a causal link between subluxations and various diseases should therefore not be made [9]. The fourth and fifth concepts deal with the treatment, namely (iv) that chiropractic adjustments can remove subluxations, (v) resulting in improved health status. However, even if there were an improvement of a condition following treatment, this does not mean that the underlying theory is correct. In other words, any improvement may or may not be caused by the treatment, and even if so, it does not automatically validate the underlying theory that subluxations cause disease.

### The duty to test non-plausible clinical activities

If PP of all sorts of diseases through chiropractic care were possible, it would of course be highly relevant both on a personal level and from a public health perspective. However, if this is not the case, chiropractors promoting this paradigm would be fooling their patients in an unethical manner. Also, when health care practitioners promote non-plausible clinical models, the burden of evidence is surely much higher than when they deal with plausible and generally acceptable concepts [10]. Therefore, it is urgently necessary to review the literature for evidence that chiropractic adjustments have an all-embracing effect on the human body, as this is not based on a plausible model.

### Confidence in poor quality research

During our readings of the literature in this domain we came across the previously mentioned review of chiropractic research [8] that had used a non-systematic and non-transparent approach in terms of search methods, inclusion and exclusion criteria, quality assessment, and synthesis of results. The author, nevertheless, concluded that chiropractic adjustments “confer measurable health benefits to people regardless of the presence or absence of symptoms” and “the notion that there is no evidence of chiropractic care being of benefit to individuals without musculoskeletal complaints appears erroneous”. With such a publication in hand, many research-naïve chiropractors would consider themselves ‘evidence-based’, when giving chiropractic adjustments with the intent to prevent disease from occurring.

### Pedagogic dimension

Presumably authors of methodologically weak reports are unlikely to perform low quality studies on purpose. It is our belief that such studies are the result of an incomplete understanding of the basic criteria for ‘good’ research and lack of assistance from the accepted research community. There is also the problem of the lack of any ‘good’ research in existence. So it becomes also a dual issue of reviewers not understanding good research and the lack of good research for them to ‘understand’. Therefore, it would be important not only to uncover the evidence level for the use of chiropractic care in PP and early secondary prevention in a literature review but also to add a pedagogic dimension to the report, in case some of the articles reviewed would be methodologically unacceptable. This expectation is not unreasonable, as – in our experience – this type of articles often seems to end up in the grey literature and in journals of lesser standard.

### Objectives

The research questions of our review were:

- For which physical, non-musculoskeletal diseases has the effect/benefit of chiropractic treatment been studied in the chiropractic literature?

And within those studies:

- Which study designs have been used?
- Were the designs appropriate to uncover effect of intervention?
- Was the basic methodological quality sufficient to make results credible?
- What evidence is there that chiropractic treatment can prevent disease or stop it in its early stage?

## Method

The review was registered in PROSPERO, with the reference CRD42017074245.

### Identifying relevant studies

We performed a number of systematic searches, scrutinizing both the ‘normal’ indexed scientific literature and the more ‘grey’ literature to which we had access. A librarian was consulted for the search of articles in journals that could be traced through *PubMed* and *Embase*. MeSH terms (PubMed) and Emtree terms (Embase) were not used because they would not necessarily exist or be helpful for this topic. Instead, we did a free text search using terms in relation to chiropractic and prevention/wellness, accepting that we would have to screen a large number of irrelevant texts in order to capture the few studies on this topic that might exist. Additional searches were performed using *the Index of Chiropractic Literature*, *Journal of Chiropractic Medicine*, *Journal of Vertebral Subluxation Research*, and the journal *Functional Neurology, Rehabilitation, and Ergonomics*. The equations and search terms are available in Table 1 (col. 1 and 2). In addition, we scrutinized the reference list of the previously mentioned review by Hannon [8] for studies on effect or benefit of treatment. A hand search was also done consulting texts and reference lists of relevant articles. We accepted only articles written in English and there was no limitation for year of publication.

**Table 1:** Search terms used in a systematic review on the effect/benefit of chiropractic primary or early secondary prevention

Journal/Online library	Search terms, issues and reference list used	Date of the last search	# articles included/# total articles
PubMed	(chiropract* OR subluxat* OR ‘manual therapy’ OR ‘spinal manipulation’ OR ‘spinal manipulative’) AND (prevent* OR wellness OR disease OR mortality OR morbidity)	04/10/2017	5 / 8628
Embase	(chiropract* OR subluxat* OR ‘manual therapy’ OR ‘spinal manipulation’ OR ‘spinal manipulative’) AND (prevent* OR wellness OR disease OR mortality OR morbidity) [embase]/lim not [medline]/ lim)	29/09/2017	1 / 2774
Index Chiropractic Literature (ICL)	Prevention (search 1)	07/10/2017	0 / 535
	Wellness (search 2)	07/10/2017	1 / 199
Journal of Chiropractic Medicine (JCM)	*All the issues from inception to 2017 were screened.*	07/10/2017	2 / 486^a^
Journal of Vertebral Subluxation Research (JVSR)	*All the issues from inception to 2017 were screened.*	07/10/2017	8 / 351^a^
Functional Neurology, Rehabilitation, and Ergonomics (FNRE)	*All the issues from inception to 2017 were screened.*	07/10/2017	0 / 126^a^
Hannon [8]	Reference list of the article: *Hannon SM. Objective Physiologic Changes and Associated Health Benefits of Chiropractic Adjustments in Asymptomatic Subjects: A Review of the Literature. J. Vertebral Subluxation Res. 2004.*	NA	3 / 65

*NA* non applicable

^a^Denominator based on number of full scientific reports excluding letters to editorials, letters to editor, etc

### Article selection

Two authors (GG and CLY) independently selected the peer-reviewed articles from the titles on *PubMed, Embase, Index of Chiropractic Literature*, and the articles obtained through our other sources. They also screened all the issues of the: *Journal of Vertebral Subluxation Research* and the *Journal of Chiropractic Medicine*. In addition, GG screened the *Functional Neurology, Rehabilitation and Ergonomics* journal twice and blindly, and the reference lists of relevant articles were searched by the same author. Potentially interesting articles were scrutinized independently by GG and CLY for inclusion and exclusion criteria using abstracts and, if in doubt, full texts.

#### Inclusion criteria

We included published research articles that suggested or specifically stated that they studied the clinical preventive *effec*t *of* or *benefit from* manipulative therapy/chiropractic treatment (with or without adjunctive measures) in relation to PP and/or early treatment of physical diseases/morbidity in general, other than musculoskeletal disorders. Also, studies including early markers of ill health were included. Included were also studies of prevention of early death. Clinical and epidemiological studies were eligible. Chiropractic treatment was defined as any treatment provided by a chiropractor. Outcome had to be studied in a clinical context, meaning that purely experimental studies with baseline and immediate post-treatment measurements were not of interest.

We referred to some case studies but did not include them in our formal analysis because we did not classify them as ‘research articles’. The reason for their inclusion was that they gave the impression to the reader that they studied some type of prevention and we wanted to deal with this from a pedagogic angle. However, they were not included in our PRISMA chart, as they had not been obtained in an exhaustive search procedure; they were rather papers that happened to be found whilst we searched for our ‘proper’ studies and were allowed to remain. Reviews, discussion papers, abstract proceedings, comments, letters to the editor, and animal studies were not included.

#### Exclusion criteria

In addition to the case study exclusion we excluded studies on risk of falling, improved sport performance, infertility and pregnancy. We excluded also studies on ‘wellness’ and general well-being as measured exclusively through questionnaires and we did not take these aspects into account if they were included as a part of an otherwise relevant study.

## Charting the data

### Checklists

Five checklists were designed in relation to the description of the reviewed articles and some basic quality criteria.

A preliminary read of some of these articles indicated that it would not be possible to perform a formal Cochrane type review for effect of treatment, due to the generally poor methodological quality. The quality control of articles was therefore simple, concentrating mainly on the study design to establish if it were at all possible to answer questions on effect of treatment in a scientifically acceptable manner. The quality checklist for the clinical studies was basic, consisting only of the major methodological points expected to be present in randomized controlled clinical trials, as this is the study design one would expect when searching for effect of treatment/intervention. Another simple checklist was used for population studies.

Our checklists have been described in Tables 2, 3, 4, 5 and 6.

**Table 2:** Description of eight clinical studies on chiropractic primary or early secondary prevention included in a systematic review

First Author (Year) Journal Affiliation Country	Research question(s) or purpose of study	Type of manipulative therapy/chiropractic treatment	Outcome variables for studied condition	Authors’/author’s conclusion in relation to effect/benefit of chiropractic treatment
Kessinger (1997) JVSR ? USA	“to assess the influence of upper cervical adjustments on pulmonary function.”	Upper cervical treatment	Lung function: a)forced vital capacity (FVC); b)forced expiratory volume in one second (FEV-1)	“The study indicates that subjects show improved pulmonary function in FVC and FEV-1 after receiving chiropractic care for the correction of upper cervical vertebral subluxation.”
Kessinger (1998) JVSR ? USA	“to investigate the relationship between frequency of adjustments (hence presence of a vertebral subluxation) and changes in visual acuity among a population of subjects previously naïve to any form of chiropractic.”	“Upper Cervical Specific Care for the correction of atlas and/or axis (C-1, C-2)”	Distance visual acuity	“This information suggests that correction of upper cervical subluxation, regardless of its vector character (right versus left, or inferior or superior to axis) is associated with either uni-lateral, and/or bilateral improvements in %DVA.” “Thus, through the upper cervical adjustment procedures employed in the present study, improvement in visual acuity appears to be linked to correction of vertebral subluxation.” “Consequently, while further study of other subject populations is required to validate the preliminary findings presented in this article, it appears that the effects observed are of longer term than would be expected from a stimulus-response reaction. Further evaluation, however, will be needed to elucidate the long term nature of the effects observed, as well as to decipher the differential vision changes apparent in the present study.” *DVA = Distance Visual Acuity*
Morter (1998) JVSR Morter Health System, Inc. and developer of Bio-Energetic Synchroniza-tion Technique USA	Testing the hypothesis that “lower salivary pH would accompany excessive sympathetic stimulation while higher pH values would accompany parasympathetic predominance”. Also to test the hypothesis that “salivary pH values would increase or decrease accordingly after administration of care”.	Bio-energetic synchronization which “updates or re-sets engrams eliciting inappropriate physiology often associated with autonomic imbalance”	Salivary pH	“Effect sizes for the two groups revealed a large treatment effect in the S-Group (0.80) compared to a moderate effect in the P-Group (0.50) …” *S-Group = sympathetic group* *P-Group = sympathetic group*
Campbell (2005) JVSR Camgen, Inc. USA	“to assess the effect of short-term and long-term chiropractic care on serum thiol levels in asymptomatic subjects” *Thiol = a surrogate estimate of human health status; of DNA repair enzyme activity*	-network spinal analysis -diversified technique with drop-table -activator methods	Plasm/serum thiol	“Asymptomatic or primary wellness subjects under chiropractic care demonstrated higher mean serum thiol levels than patients with active disease and produced some values that were higher than normal wellness values.” “chiropractic could influence the basic physiological process of endogenous generation of oxidative stress”
Boone (2006) JVSR Sherman College of Straight Chiropractic USA	Pilot study “to gather preliminary information regarding chiropractic care and possible links to immune status and improved aspects of health and quality of life”	-“chiropractic care” ***-***“chiropractic adjustments when indicated”	Blood tests for immunological markers	“This pilot study has provided some preliminary information regarding chiropractic care and possible links to immune status …”
McMasters (2013) Journal of Chiropractic Medicine Private practice USA	“to determine if a course of chiropractic care would change BP measurements in African American patients and to determine if a study was feasible in a chiropractic teaching clinic.” *BP = blood pressure*	“chiropractic adjustments (manipulation) based upon the spinal examination findings”	Systolic and diastolic blood pressure on subjects diagnosed with prehypertension or stage 1 hypertension	“There was no statistically significant difference in BP following chiropractic care for this group of African American patients. However, when 4 patients who had large BMIs (outliers) were excluded from the group, a statistically significant decrease in diastolic BP was observed. It is possible that patients with higher BMI may be more resistant to BP reductions in the context of chiropractic care. Unfortunately, the mechanism between BMI and BP is not well understood.” *BMI=Body Mass Index*
Jones (2014) Disability and Rehabilitation School of Health Science and Social Care UK	“to investigate the hypothesis that MT produces additional benefit when compared with breathing retraining alone in a group of patients with primary DB.” *MT = Manual Therapy* *DB = Dysfunctional breathing*	All the subjects were treated with standardised respiratory physiotherapy management The intervention group: individualised selection of manual therapy techniques (e.g. Maitland mobilisation/ manipulation)	***Primary outcome*** -Nijmegen score for dysfunctional breathing ***Secondary outcome*** -Spirometry measured by: a)forced expiratory volume in one second (FEV1) b)forced vital capacity (FVC); -Breath hold time	“There was no significant difference between the manual therapy and respiratory treatment groups for the primary outcome (Nijmgen score) or any secondary outcomes”
Goertz (2016) JMPT Palmer college of Chiropractic USA	Pilot study “to estimate the treatment effect and safety of toggle recoil spinal manipulation for blood pressure management”	Toggle recoil spinal manipulation therapy	Systolic and diastolic blood pressure on subjects diagnosed with prehypertension or stage 1 hypertension	“...there is limited research to support the use of SMT for patients with high BP. Thus, rigorous studies to evaluate the efficacy and safety of SMT for hypertension are needed to guide chiropractic clinical practice.” *BP=Blood Pressure* *SMT = Spinal Manipulative Therapy*

*JVSR* journal of vertebral subluxation research

*JMPT* journal of manipulative and physiological therapeutics

**Table 3:** Qualitative checklist of eight clinical studies on chiropractic primary or early secondary prevention

First Author (Year) Journal Affiliation Country	Methodological considerations					Were differences between groups tested for statistical significance in relation to effect/benefit of treatment?	Comments by reviewers in relation to major methodological improvements needed to test effect/benefit of intervention
	Design	Comparison with non-treated (placebo) group or an otherwise treated group?	Random and concealed allocation to treatment groups	Main outcome variable(s) validated in some way? (reproducible/reliable)	Assessor blinded to treatment group?		
Kessinger (1997) JVSR ? USA	Prospective outcome study of lung function after 2 weeks of chiropractic care	No placebo or control group	NA because no control group	FEV-1 reported to be most reproducible of the two measurements with ref. provided but level of reproducibility not reported	NA There was only one group	NA There was only one treatment group	To test the effect on pulmonary function after chiropractic care of the neck, one could a) either compare it to a sham treatment, or to b) another type of treatment known to be effective or (possibly) to c) a treatment elsewhere in the spine, if the purpose is to see if the ‘neck’ is important.
Kessinger (1998) JVSR ? USA	Prospective outcome study of visual acuity after six weeks of chiropractic. Also the dose of treatment was studied.	No placebo or control group	NA because no control group	Not reported but used standard eye chart	NA There was only one group	NA There was only one treatment group	To test if the ‘dose’ of adjustments matter, patients should at baseline be randomly allocated into one of several groups each receiving different numbers of treatments/adjustment.
Morter (1998) JVSR Morter Health System, Inc. and developer of Bio-Energetic Synchronization Technique USA	Prospective outcome study of salivary pH in two groups defined as predominantly sympathetic or parasympathetic after 4-days of chiropractic treatment	No placebo or control group but patients were all treated in the same way and outcomes were compared in relation to whether they predominantly were sympathetic or parasympathetic	NA because study sample stratified on predetermined criteria	Not reported but used standard pH paper	NA There was only one group	NA There was only one treatment group	To establish if different subgroups react differently to the chiropractic treatment, then the groups could either a) be tested for outcome in a randomized controlled clinical trial design or (possibly) b) be tested for outcome in a sufficiently large non-controlled prospective outcome study that allows for subgroup analyses. The diagnosis of predominantly sympathetic and parasympathetic subjects must be valid and/or reproducible. The assessment should be done with valid/reproducible methods by assessors that are blinded to classification group.
Campbell (2005) JVSR Camgen, Inc. USA	A retrospective study comparing serum thiol levels in patients with active disease (? Abstract)/apparently disease free (? Materials and Methods) for two groups (? Materials and Methods) or perhaps three groups (? Table 1). These groups had been treated with chiropractic care for a) less than one year or b) at least one year. Perhaps there was also a third non-symptomatic apparently healthy control group (? Table 1).	Perhaps, not clear	No	Serum thiols claimed to be valid as indicators for mortality and active disease	NA There was only one group	Yes	To test if chiropractic care and dose of care can affect DNA repair then a study sample should have been randomly divided into treated and untreated, and this could have been done for different study populations, the sick and the healthy. The dose-response should be tested in a similar way, i.e. a group of patients receiving short-term and one long-term treatment in a random fashion. The results in this study relate only to association and not effect. The claim that “results clearly support” etc. are unfounded.
Boone (2006) JVSR Sherman College of Straight Chiropractic USA	Prospective outcome study of immune status and health after three and nine months of chiropractic care	NA because no control group	NA because no control group	Not reported	NA There was only one group	NA There was only one treatment group	Just because a study sample is small, does not justify to call it a ‘pilot study’. A pilot study should be used to test study procedures, ability to obtain patients, etc. To draw any (even preliminary) conclusions on effect/benefits of treatment, a sham/control treatment is needed.
McMasters (2013) Journal of Chiropractic Medicine Private practice USA	Prospective outcome study of blood pressure after 21 to 23 chiropractic consultations	NA because no control group	NA because no control group	Probably valid	NA There was only one group	NA There was only one treatment group	As this is a feasibility study, it is not really appropriate to concentrate the discussion on ‘improvement’ but should concentrate more on reasons for/against the possibility to perform a proper randomized controlled trial (RCT). To test the effect of spinal manipulation on blood pressure an RCT with a sham group would be necessary.
Jones (2014) Disability and Rehabilitation School of Health Science and Social Care UK	2-arm randomized controlled trial of dysfunctional breathing after 2, 4, 8, 12 and 26 weeks of either a) respiratory management (RM) or b) RM plus manual therapy	Yes, with a control group	Yes	Yes, for the questionnaire. The other variables are frequently used so probably valid.	Yes	Yes	The design is appropriate for testing difference in outcome between treatment groups, in this case to see if manual therapy can provide added benefit to another treatment.
Goertz (2016) JMPT Palmer college of Chiropractic USA	2-arm randomized controlled trial of blood pressure after 1, 6 and 12 visits of spinal manipulation	Yes, with a sham group	Yes	Probably valid	Yes	Yes	The design is appropriate for testing whether spinal manipulation has an effect on blood pressure. However, the absence of effect should be discussed more clearly.

*JVSR* journal of vertebral subluxation research

*JMPT* journal of manipulative and physiological therapeutics

*NA* non applicable

**Table 4:** Descriptive checklist of five population studies on chiropractic primary or early secondary prevention

First Author (Year) Journal Affiliation Country	Research question(s) or purpose of study	Design	Study population	Outcome variables	Which factors associated with cause were included?	Authors’/author’s conclusion in relation to effect/benefit of chiropractic treatment
Hart [19] (2007) JVSR Sherman College of Straight Chiropractic USA	“to better understand possible mechanisms for the health disparity along the River” *Mississippi River*	Register study	General population from the states along the Mississippi River	Various diseases and mortality	Only correlations between risk factor (physician/chiropractor ratios) and outcomes (various health conditions and death) were studied	“Chiropractors had stronger correlations for improved health outcomes when compared to physicians. Further study is indicated into other possible causative mechanisms such as the quality of drinking water and health care delivery.”
Hart [20] (2007) Journal of Chiropractic Medicine Sherman College of Straight Chiropractic USA	“This study assesses doctor (allopathic/ osteopathic physician and chiropractor) ratios in the 50 states in the United States and correlates these ratios with various health outcomes to determine if one doctor type has stronger correlations in certain outcomes compared with the other doctor type by geographic region.”	Register study	General population from 50 states in the United states	Various diseases and mortality	Only correlations between risk factor (physician/chiropractor ratios) and outcomes (various health conditions and death) were studied	“Correlation does not necessarily show causation but may provide clues. […] It is possible, although care should be taken to avoid overspeculation, that doctors of chiropractic are having an effect in seemingly unlikely outcomes such as cardiovascular and cancer deaths”
Hart [21] (2008) JVSR Sherman College of Straight Chiropractic USA	As above (Hart, 2007) [20], but adding the variables of income, education and health insurance coverage in the analysis	Register study	General population from 50 states in the United states + district of Columbia	Various diseases and mortality	Only correlations between risk factor (physician/chiropractor ratios) and outcomes (various health conditions and death) were studied	“Correlation does not necessarily show causation but it can provide clues. Median income, educational attainment, and chiropractor ratios showed the strongest correlation with reduced mortality rates while health insurance and medical doctor ratios showed the weakest correlation with reduced mortality rates.”
Hart [22] JVSR (2008) JVSR Sherman College of Straight Chiropractic USA	As above (Hart, 2007) [20] but adding the variables of age, income and education in the analysis	Register study	General population from 50 states in the United states + district of Columbia	Various diseases and mortality	Only correlations between risk factor (physician/chiropractor ratios) and outcomes (various health conditions and death) were studied	“The age factor […] had the strongest association with death rates” compared to doctor ratios. “The only statistically significant relationship among doctor ratios was observed with medical doctors and cerebrovascular, though chiropractor ratios showed a stronger average correlation with reduced death rates.”
Hart [23] International Dose-Response Society (2013) Sherman College of Chiropractic USA	“to simply compare the correlation between DC and MD concentrations (doses) in relation to hypertension mortality rates (responses).” *DC = Doctors of Chiropractic* *MD = Medical Doctors*	Register study	General population from district of Columbia (without Alaska and Wyoming)	Hypertension Death rates	Only correlations between risk factor (physician/chiropractor ratios) and outcomes (various health conditions and death) were studied	“DC concentrations (dose) revealed a stronger beneficial correlation with decreased hypertension (essential hypertension and renal hypertensive disease) mortality rates (response) compared to MD concentrations” (Causal inference is not claimed.)

*JVSR* journal of vertebral subluxation research

**Table 5:** Qualitative checklist of five population studies on chiropractic primary or early secondary prevention

First Author (Year) Journal Affiliation Country	Representativeness		Definition of chiropractic treatment	Outcome variables validated in some way?	Control for other variables that could have an effect on outcome	Comments by reviewers in relation to major methodological improvements needed to test effect/benefit of intervention
	Selection of study subjects (whole population, random selection, convenience sample)	Response/ Non response comparison				
Hart [19] (2007) JVSR Sherman College of Straight Chiropractic USA	Whole population?	Not reported	Chiropractic care not described Presence of chiropractors	Probably acceptable, official register	No	To examine if chiropractors as opposed to medical practitioners have a real effect on health outcomes on a public health level, a more sophisticated type of analysis would be needed, taking into account a large number of variables that are linked to both the relative presence of chiropractors and the development of disease. This would have to be tested in multivariate models as it is not enough to investigate such variables one by one holding them up against the outcome variables (e.g. disease or mortality rates).
Hart [20] (2007) Journal of Chiropractic Medicine Sherman College of Straight Chiropractic USA	Whole population?	Not reported	Chiropractic care not described Presence of chiropractors	Probably acceptable, official register	No	See above
Hart [21] (2008) JVSR Sherman College of Straight Chiropractic USA	Whole population?	Not reported	Chiropractic care not described Presence of chiropractors	Probably acceptable, official register	No	See above
Hart [22] JVSR (2008) JVSR Sherman College of Straight Chiropractic USA	Whole population?	Not reported	Chiropractic care not described Presence of chiropractors	Probably acceptable, official register	No	See above
Hart [23] International Dose-Response Society (2013) Sherman College of Chiropractic USA	Whole population?	Not reported	Chiropractic care not described Presence of chiropractors	Not explained	No	See above

*JVSR* Journal of Vertebral Subluxation Research

**Table 6:** Descriptive checklist of five case studies on chiropractic primary or early secondary prevention

First Author (Year) Journal Affiliation Country	Disorder studied	Type of treatment	Authors’/author’s conclusion in relation to effect/benefit of chiropractic treatment
Blum (2006) JVSR Private Practice USA	Early onset diabetes mellitus	-sacro-occipital technique -occipital fiber diagnosis and treatment -bloodless surgery -chiropractic Manipulative Reflex Technique -also: dietary modifications and exercise	“Within one month of treatment his glucose blood and urine levels had normalized and remained stable.”
Fedorchuk (2011) AVSR Private Practice USA	Cholesterol levels	-diversified technique -active Release technique -chiropractic biomechanics of posture techniques (CBP)	“The clinical process documented in this report suggests that the combination of Diversified and CBP chiropractic care reduces subluxations and the tensegrity stress on the spinal column and nervous system. As a result of this reduced stress there is reduction of dysponesis which is evidenced by the improved quality of life and blood serum cholesterol levels.”
Zielinski (2013) AVSR Life University College of Chiropractic, Emory University School of Public Health USA	Multiple conditions in a patient with dyslipidemia	-no life-style changes -torque release technique -diversified technique on C1 and sacrum/pelvis	“As care progressed, patient’s subjective stress levels decreased. […] We suspect his lipid levels were normalized as a consequence of decreased stress and subsequent normalizing in cortisol and inflammatory factors.”
Slinger (2014) AVSR Private Practice USA	Cardiovascular disease risk factors	-diversified technique -lifestyle changes (diet and exercise)	“This retrospective case study reports on the effectiveness of chiropractic care in reducing vertebral and lower extremity subluxation findings as well as lowering the risk factors of cardiovascular disease” (serum cholesterol and lipid panels)
Knowles (2015) AVSR Private Practice USA	Heart rate variability (as a proxy for a healthy state)	Network spinal analysis care	“After 6 months of Network care, follow-up examinations were performed: heart rate variability, […]. Surface EMG demonstrated an improvement in all areas of tension exhibited at the initial exam” *EMG = Electromyography*

*JVSR* journal of vertebral subluxation research

*AVSR* annals of vertebral subluxation research

1. Descriptive checklist for clinical studies (Table 2):

- First author, year of publication, name of journal, affiliation, country;
- Research question(s)/purpose of the study;
- Type of manipulative therapy/chiropractic treatment;
- Outcome variables for studied condition;
- Authors’/author’s conclusion in relation to effect/benefit of chiropractic treatment.

Quality checklist for clinical studies (Table 3):

- Methodological considerations
- Design
- Comparison with non-treated (placebo) or an otherwise treated group;
- Random and concealed allocation to treatment groups;
- Main outcome variable(s) validated in some way;
- Assessor blinded to treatment group.
- Were differences between groups tested for statistical significance in relation to effect/benefit of treatment?
- Comments by reviewers in relation to major methodological improvements needed to test effect/benefit of intervention.

2. Descriptive checklist for population studies (Table 4):

- First author, year of publication, name of journal, affiliation, country;
- Research question(s)/purpose of the study;
- Design;
- Study population;
- Outcome variables;
- Which factors associated with cause were included?
- Authors’/author’s conclusion in relation to effect/benefit of chiropractic treatment.

Quality checklist for population studies (Table 5):

- Selection of study subjects (whole population, random selection, convenience sample);
- Response/Non response comparison;
- Definition of chiropractic treatment;
- Outcome variables validated in some way;
- Control for other variables that could have an effect on outcome;
- Comments by reviewers in relation to major methodological improvements needed to test effect/benefit of intervention.

Finally, a descriptive checklist (Table 6) was designed for case studies on this subject in order to have an overview of the diseases studied in the chiropractic literature.

The items were:

- First author, year of publication, name of journal, affiliation, country;
- Disorder studied;
- Type of treatment;
- Authors’/Author’s conclusion in relation to effect/benefit of chiropractic treatment.

#### Data extraction and analysis

Data extraction was made independently by GG and CLY with the possibility to consult the third author in case of continued disagreement. Data interpretation was done through discussions. Each article was discussed in view of its checklist findings and any methodological weaknesses. Agreement had also to be reached on which major remedial action would have been relevant for the various types of studies, if their quality was considered substandard, in relation to determining effect/benefit of treatment. We did not check contents of references to trace additional or missing information.

## Results

### Descriptive information

**Fig. 1:**
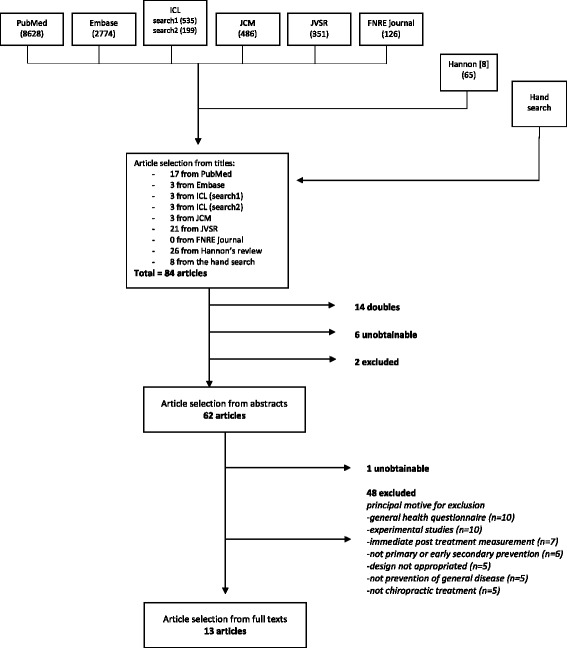
Description of the search for literature in a review on chiropractic primary and/or early secondary prevention. ICL = Index of Chiropractic Literature. JCM = Journal of Chiropractic Medicine. JVSR = Journal of Vertebral Subluxation Research. FNRE = Functional Neurology, Rehabilitation, and Ergonomics. Review by Hannon [8]

Of the 13.099 titles scrutinized, we retained 13 full text articles for the final review (Fig. 1). Of these, five articles came from our *PubMed/Embase* searches, whilst eight were found in the *Journal of Vertebral Subluxation Research*. As described in Tables 2 and 3, eight were prospective clinical studies [11–18] published between 1997 and 2016. Seven originated in America and six were published in the *Journal of Vertebral Subluxation Research*. Five register studies [19–23] were included (Tables 4 and 5), published between 2007 and 2013 by the same author. Three out of these five articles were published in the *Journal of Vertebral Subluxation Research*. Finally, we included five case reports [24–28] that were found in the reference list of Hannon’s review (Table 6). These all originated in America and were also published in the *Journal/Annals of Vertebral Subluxation Research.* Six potentially relevant studies in Hannon’s review and one from an additional hand search could not be obtained (Table 7). However, only two of these appeared to be of interest because the titles included the word ‘effect’. The provenance of all the selected articles and the case studies is illustrated in Table 8.

**Table 7:** Studies on effect/benefit of chiropractic primary or early secondary prevention that could not be obtained in a systematic review

References	Hannon [8]	Hand search
Vora GS, Bates HA. The effect of Spinal Manipulation on the Immune System (A Preliminary Report). ACA Journal of Chiropractic. 1980;14:S103–105.	X	
Masarsky CS, Weber M. Chiropractic and Lung Volumes – A retrospective Study. ACA Journal of Chiropractic. 1986;20(9):65–67.	X	
Lott GS, Sauer AD, Wahl DR, Kessinger J. ECG Improvements Following the Treatment Combination of Chiropractic Adjustments, Diet, and Exercise Therapy. The Journal of Chiropractic Research and Clinical Investigation. 1990;6(2):37–39.	X	
Hoiriis KT, Owens EF, Pfleger B. Changes in general health status during upper cervical chiropractic care: A practice-based research project. Chiropractic Research Journal. 1997;4(1):18–26.		X
Owens EF, Hoiriis KT, Burd D. Changes in General Health Status During Upper Cervical Chiropractic Care: PBR Progress Report. CRJ. 1998;5(1):9–16.	X	
Kessinger R, Boneva D. Neurocognitive Function and the Upper Cervical Spine. CRJ. 1999;6(2):88–89.	X	
Miller JA, Bulbulian R, Sherwood WH, Kovach M. The Effect of Spinal Manipulation and Soft Tissue Massage on Human Endurance and Cardiac and Pulmonary Physiology – A Pilot Study. The Journal of Sports Chiropractic & Rehabilitation. 2000;March:11–15	X

**Table 8:** Provenance of included articles in a systematic review on chiropractic primary and/or early secondary prevention

	Articles	PubMed	Embase	ICL search 2	JCM	JVSR	Hannon [8]
Clinical studies	Kessinger [11] (1997)					X	X
	Kessinger [12] (1998)					X	X
	Morter [13] (1998)					X	X
	Campbell [14] (2005)			X		X	
	Boone [15] (2006)					X	
	McMaster [16] (2013)	X			X		
	Jones [17] (2014)	X					
	Goertz [18] (2016)	X					
Population studies	Hart [19] (2007)					X	
	Hart [20] (2007)	X			X		
	Hart [21] (2008)					X	
	Hart [22] (2008)					X	
	Hart [23] (2013)	X	X				
Case studies	Blum [24] (2006)					X	
	Fedorchuk [25] (2011)					X	
	Zielinski [26] (2013)					X	
	Slinger [27] (2014)					X	
	Knowles [28] (2015)					X	

*ICL* index to chiropractic literature

*JCM* journal of chiropractic medicine

*JVSR* Journal of Vertebral Subluxation Research

Review by Hannon [8]

### Reasons for inclusion in the review

Words used to indicate that the investigator or reader would have in mind effect or benefit of treatment when reading these articles were: “influence”, “relationship”, “increase...after care”, “assess effect”, “possible links to ... improved aspects of”, “change”, “produces additional benefits”, and “treatment effect”.

### For which physical, non-musculoskeletal diseases has the effect/benefit of chiropractic treatment been studied in the chiropractic literature?

Disorders studied in the clinical studies were: lung function (*n* = 2), systolic and diastolic blood pressure (*n* = 2), visual acuity, and three proxies for/markers of good health: salivary pH, plasma serum thiol, and blood test immunological markers (Tables 2 and 3).

The five population studies (Tables 4 and 5) dealt with various disorders of public health importance, such as obesity, infectious disease, but also mortality.

The five case reports dealt with diabetes mellitus, cholesterol levels, multiple conditions with dyslipidaemia, cardiovascular disease risk factors. Heart rate variability was studied as a proxy for/marker of a healthy state (Table 6).

### Which study designs have been used and were they relevant to uncover effect or benefit of intervention?

Although most clinical studies were prospective in design (Tables 2 and 3) they were mainly simple descriptive outcome studies without control group. Three included a control group but only two used a *prospective* two-arm randomized controlled study design; one including two identically treated groups with spinal manipulation added to one of these (studying added benefit of treatment) [17], and one consisting of a treated and a placebo group (studying effect of treatment) [18]. The third study with a control group used a retrospective case-control study design in relation to the influence of chiropractic treatment on DNA repair [14]. Probably, only clinical studies using a prospective two-arm approach would be able to compare outcomes between different treatments.

Also, properly conducted population studies could be able to cast some light on the benefit of chiropractic care from a public health perspective. The five included population studies obtained their data from N. American national registers, which might be interesting depending on the origin, completeness, contents, and quality of data of these registers.

The case reports (Table 6) consisted of particular patients encountered in clinical practice probably with retrospective data collection. Case reports are usually not suitable to answer research questions relating to treatment effect.

Therefore, theoretically, seven or possibly eight of the articles would be able to provide answers relating to effect and/or benefit of manipulative therapy/chiropractic care in the area of prevention and early treatment of disease, namely two or three clinical studies that included a control group [14, 17, 18] and five population studies [19–23]. Nevertheless, a further look at the study approach of the retrospective clinical study and the population studies revealed considerable weaknesses.

### Was the basic methodological quality sufficient to make results credible?

#### Clinical studies

Three of the clinical studies included a control group, whether placebo or other treatment. However, the study that was retrospective in design was unlikely to have included objectively studied and similar patients, which makes comparisons of treatment outcomes non-credible. Therefore, only the prospective studies had the potential of providing correct answers to their research questions on effect and benefit. Both these studies included also important aspects such as blinding of patients (in the effect-study) and blinding of assessors (in both studies) and described carefully their study methods and results in a transparent manner, allowing for reproduction of their studies. The retrospective study was not set up as an experiment and therefore did not use blinded assessors. In addition, methodological information was missing and confusing in the report.

#### Population studies

None of the register studies described clearly the origin and quality of these registers (Tables 4 and 5) and, in the analysis, did not take into account the complex correlations between potential and known moderating and confounding factors in real life that have an effect on morbidity and mortality and were therefore not able to account for the potential independent influence that medical and/or chiropractic practitioner density could have on health outcomes.

#### Case reports

As previously mentioned, case-reports are not suitable to describe effect or benefit of treatment. Despite this, one of the authors [26] wants to promote the idea that subluxation-based chiropractic care may help prevent heart disease and various other conditions.

### What evidence of effect is there?

In sum, we found no evidence of effect of manipulative therapy/chiropractic treatment based on these included articles. Only one article can be used to draw conclusions on ‘effect’. The prospective two-armed randomized controlled clinical trial compared a real treatment on a ‘treated group’ (i.e. toggle recoil) with a sham treatment in a ‘placebo group’, on subjects diagnosed with prehypertension or stage 1 hypertension. According to this article, there was an absence of effect of spinal manipulation therapy.

The other methodologically acceptable article reported on a prospective two-armed randomized controlled clinical that was suitable to determine if manipulative therapy had an ‘added benefit’ in patients with primary dysfunctional breathing, who all received breathing retraining. No such benefit was noted.

None of the other articles were considered suitable to establish effect or benefit of chiropractic treatment.

## Discussion

### Summary

Of the approximatively 13.000 articles initially identified as potentially suitable for our review only 13 were included. Although their authors usually avoided to state explicitly that they were studying effect of treatment, they would imply somehow in their text that this was the case, which earned them a place in our review. A wide variety of diseases were examined but, according to the only two studies with a suitable research design to answer our research questions, there was no effect or benefit of chiropractic care in the prevention/early treatment of high blood pressure and no extra benefit was found for manipulative therapy on dysfunctional breathing. In sum, we could not find any evidence in favour of the argument that manipulative therapy/chiropractic care can prevent or stop early disease.

### General methodological considerations

Although at first look there appears to be a literature on this subject, it is apparent that most authors lack knowledge in research methodology. The two methodologically acceptable studies in our review were found in *PubMed*, whereas most of the others were identified in the non-indexed literature. We therefore conclude that it may not be worthwhile in the future to search extensively the non-indexed chiropractic literature for high quality research articles.

One misunderstanding requires some explanations; case reports are usually not considered suitable evidence for effect of treatment, even if the cases relate to patients who ‘recovered’ with treatment. The reasons for this are multiple, such as:

- Individual cases, usually picked out on the basis of their uniqueness, do not reflect general patterns.
- Individual successful cases, even if correctly interpreted must be validated in a ‘proper’ research design, which usually means that presumed effect must be tested in a properly powered and designed randomized controlled trial.
- One or two successful cases may reflect a true but very unusual recovery, and such cases are more likely to be written up and published as clinicians do not take the time to marvel over and spend time on writing and publishing all the other unsuccessful treatment attempts.
- Recovery may be co-incidental, caused by some other aspect in the patient’s life or it may simply reflect the natural course of the disease, such as natural remission or the regression towards the mean, which in human physiology means that low values tend to increase and high values decrease over time.
- Cases are usually captured at the end because the results indicate success, meaning that the clinical file has to be reconstructed, because tests were used for clinical reasons and not for research reasons (i.e. recorded by the treating clinician during an ordinary clinical session) and therefore usually not objective and reproducible.
- The presumed results of the treatment of the disease is communicated from the patient to the treating clinician and not to a third, neutral person and obviously this link is not blinded, so the clinician is both biased in favour of his own treatment and aware of which treatment was given, and so is the patient, which may result in overly positive reporting. The patient wants to please the sympathetic clinician and the clinician is proud of his own work and overestimates the results.
- The long-term effects are usually not known.
- Further, and most importantly, there is no control group, so it is impossible to compare the results to an untreated or otherwise treated person or group of persons.

Nevertheless, it is common to see case reports in some research journals and in communities with readers/practitioners without a firmly established research culture it is often considered a good thing to ‘start’ by publishing case reports.

Case reports are useful for other reasons, such as indicating the need for further clinical studies in a specific patient population, describing a clinical presentation or treatment approach, explaining particular procedures, discussing cases, and referring to the evidence behind a clinical process, but they should not be used to make people believe that there is an effect of treatment. In fact, there are ‘rules’ for how to deal with case reports, such as the CARE guidelines by Gagnier et al. [29].

All clinical studies but two suffered from serious methodological problems.

- The main problem was that five out of the eight prospective outcome studies did not have a control group. Clearly, in order to find out if a treatment has an effect*,* a comparison to no treatment must be made or a comparison to another treatment that is known to have an effect. Further, this ‘no treatment’ group must be masked into a sham treatment, to allow for the placebo effect that probably always plays a role in clinical practice.
- Interestingly, only two of the five prospective studies without a control group mentioned this as a problem. Nevertheless, instead of discussing this lack of control group, the authors of three articles mentioned that there would be a need for larger studies. However, larger studies will not remedy this fundamental flaw in the study design.
- When comparing outcomes between different types of treatment approach, the sham group is not relevant but the study subjects should not have a preference for one type of treatment or the other. Therefore, it is difficult to perform such studies on chiropractors, chiropractic patients and chiropractic students, as study participants should be ‘naïve’. To account for expectation bias, study subjects’ preferences should be elicited prior to the start of the study and taken into account during the analysis.
- When establishing effect or benefit of treatment, it is also necessary that the study subjects are captured at about the same period of time, as the disease, the treatment and study subjects may change over time. It is usually not a good idea to simply compare one type of treatment with the results of another type of treatment carried out x number of years ago or at the same time in some other clinic. The reasons why the study subjects should be captured in the same place is that they should be fairly representative of that patient group, and different countries, areas of a country, clinics and clinicians may attract fundamentally different types of patients with inherently different prognoses.
- The allocation into one study group or another should be done in a random fashion, in such a way that nobody can guide different patient types into a specific group because they seem more suitable in that group. Random allocation usually avoids clustering of certain patient types in one group, which may have an effect on treatment outcome if these groups react differently to treatment.
- Other important aspects are that the person who assesses the outcome should not be the person who treats the patient and should also be blind to which study group the person assessed belongs to. The outcome variables should be objective and relevant in relation to measuring whether the disease improved or not. Further, tests should be reliable when carried out by different examiners and also consistent (reproducible) within the study subject if the test is carried out over several times, to ensure that any changes occurring over time are due to the treatment and not to the instability of the test or inability of the tester.

Theoretically, it would be possible in population-based studies to compare patterns of disease and mortality in relation to various factors, such as the density of various health practitioners in the population. This could be done in epidemiologic studies of randomly selected people from the general population, ecological studies in which people live through a so-called ‘natural experiments’, and data bases from the health insurance industry or those holding socio-economic, morbidity and mortality data.

However, to observe the ‘effect’ of chiropractic care, through a comparison of an area with access to chiropractic care versus an area with less or no such access, as was the attempt in the five reviewed population studies, it would be necessary to take into account factors that might incite chiropractors to settle in a specific area. In poorer areas there would be fewer chiropractors because of the difficulty to run a successful practice but poor people are also sicker and die younger than more financially comfortable people [30, 31]. Therefore, increased morbidity or mortality that seems to be linked with the number of chiropractors would instead depend on other more fundamental factors. None of the five register studies included in this review took into account, properly, the moderating or confounding influence that such variables could have on their initial results. They simply reported the links between chiropractor density and various other predictive factors vs. disease or mortality in independently reported analyses, such as comparing the number of deaths in relation to a) the mean age of the study sample, b) the type of social class in the area, c) the proportion of chiropractors vs. general practitioners but did not combine these using appropriate statistical methods.

In comparison, we reviewed a population-based study on the elderly population in N. America [32] but could not include it because the outcome was established through questionnaires, not through objective measurements. This study collected a large number of variables at baseline on community dwelling persons who went through a clinical trial. They then compared health outcomes for those who consulted chiropractors and those who did not, and found that there seemed to be a better health profile for the chiropractic subgroup. However, when they statistically controlled on a number of base-line variables which indicated that the two groups were somewhat different (in relation to age, strenuous physical activity, health status, and arthritis status), the difference between the chiropractic group and the other disappeared. The explanation for this is that these additional factors were associated to both the choice of practitioner and the health outcome. However, *they* were the reasons for health and disease, not the health practitioner. Therefore, the link between the use of chiropractors and better health was only an apparent one. This example is given to illustrate the importance of including relevant ‘competing’ factors when looking at cause-effect in population studies.

### Methodological consideration of the review process

The screening of so many titles may result in errors due to fatigue but it was done blindly by two of the authors to avoid mistakes and it was never necessary to consult the third author. In relation to the journal *Functional Neurology, Rehabilitation and Ergonomics*, in which all published articles were screened, this was done by only one of the authors but it was done blindly at two separate occasions. Although some studies could not be found, it is unlikely that they would have brought any positive evidence for chiropractic care and PP or early secondary prevention, as they were published in the ‘non-indexed’ literature in *PubMed*.

We designed our own checklists to meet the specific needs of this review. It was not considered appropriate to employ the Cochrane checklist, for example, as preliminary readings of some of this literature indicated that the quality problems would become apparent with a much less sophisticated scrutiny. Another research team would probably have designed a somewhat different list of items. However, this would undoubtedly have identified the glaring methodological problems apparent in most of this literature. Our checklists were easily completed, and the third reviewer was, again, not required to act as a referee, indicating the checklists were user-friendly. The results were interpreted in a narrative way, no meta-analysis has been done, because this was not appropriate.

We reviewed only 13 relevant articles but there is, in fact, a literature on the experimental effects of spinal manipulation in relation to various physiological outcome variables. These were not included, as we were interested in the clinical picture only. Also, the review approach to such articles would have to be different and should therefore be done in separate reviews.

### Future considerations

#### The need for evidence

For groups of chiropractors, prevention of disease through chiropractic treatment makes perfect sense, yet the credible literature is void of evidence thereof. Still, the majority of chiropractors practising this way probably believe that there is plenty of evidence in the literature. Clearly, if the chiropractic profession wishes to maintain credibility, it is time seriously to face this issue. Presently, there seems to be no reason why political associations and educational institutions should recommend spinal care to prevent disease in general, unless relevant and acceptable research evidence can be produced to support such activities. In order to be allowed to continue this practice, proper and relevant research is therefore needed. However, such activities need to be guided by some fundamental concepts, as discussed below.

#### First, the concept of biological plausibility

In order to proceed to a research study, there must be a credible anatomical, physiological, and/or biological rationale for the link between the treatment and the PP or early secondary prevention of the disease under scrutiny.

#### Second, the concept of quality of research

In order to show effect of intervention, properly conducted randomized controlled trials should be carried out, as described above. This usually requires the participation of independent and professional researchers and they are costly and therefore require funding. Further, it is unethical to conduct poor quality studies because: they inconvenience subjects on studies with no consequences, they are a waste of money that could have been used on quality projects, and they can be misleading for both chiropractors and their patients. High quality, honest studies evoke admiration and acceptance in scientific and health care environments and will have a good effect on the chiropractic profession, for everybody to enjoy, regardless if the results are ‘positive’ or ‘negative’.

#### The concept of the three pillars of evidence based practice

The three pillars of evidence-based medicine are often described as (i) the scientific evidence, (ii) the practitioner’s experience, (iii) and the patient’s preference. However, the practitioner’s experience is not objective (please see the description of the problems with case reports above), in particular in relation to ‘effect’ of treatment.

It is therefore not enough to say that ‘it works’. The clinical experience is important in many other ways but not for judging effect of treatment. Therefore, as it has been stated before [33], “it is important to keep a humble attitude to one’s own clinical experience and not to think that it overrides the evidence obtained in a good quality RCT”.

### The need for educating chiropractors on how to read and evaluate research

All chiropractors who want to update their knowledge or to have an evidence-based practice will search new information on the internet. If they are not trained to read the scientific literature, they might trust any article. In this situation, it is logical that the ‘believers’ will choose ‘attractive’ articles and trust the results, without checking the quality of the studies. It is therefore important to educate chiropractors to become relatively competent consumers of research, so they will not assume that every published article is a verity in itself.

### Prevention in chiropractic practice

The desire to improve health in general for patients [3] indicates a common idealistic streak in large groups of the chiropractic profession. In relation to illness in general, all clinicians have a duty to inform and assist patients to avoid preventable disorders, and chiropractors can provide inspiration in this area and also monitor lifestyle changes, as back pain is a recurring disorder that often results in long term clinical relationships. However, prevention of disease through spinal adjustment is, until further notice, futile.

## Conclusion

We could find no evidence in the literature for chiropractic care as a credible approach to primary prevention or early secondary prevention in general health.

Chiropractors should therefore assume their responsibilities as an evidence-based, mature health care profession and seize such activities until, if ever, new evidence emerges.

Prevention can still be an important part of the chiropractic scope, if it is aimed at well researched and acceptable approaches such as the management of lifestyle factors to prevent co-morbidities so often found together with spinal problems.

Back problems are highly prevalent in the population and as they are usually recurring disorders there is a need for a knowledgeable and realistic profession to care for people during acute episodes and to guide them through the various periods of back pain.

It is unclear why some chiropractors feel the need to extend their scope of practice into implausible areas, when there is so much to do in the musculoskeletal field.

## Abbreviation

PP: primary prevention
